# Targeting myeloid cells for hematological malignancies: the present and future

**DOI:** 10.1186/s40364-025-00775-1

**Published:** 2025-04-10

**Authors:** Zihui Guan, Zhengqi Zhang, Kaiyan Wang, Shukai Qiao, Teng Ma, Lina Wu

**Affiliations:** 1 Key Laboratory of Carcinogenesis and Translational Research (Ministry of Education/Beijing), Central Laboratory, Peking University Cancer Hospital & Institute, Beijing, 100142 China; 2 Peking University First Hospital, Beijing, 100034 China; 3 Department of Hematology, the Second Hospital of Hebei Medical University, Shijiazhuang, 050000 Hebei China; 4 Cancer Research Center, Beijing Tuberculosis and Thoracic Tumor Research Institute, Beijing Chest Hospital, Capital Medical University, Beijing, 101149 China

**Keywords:** Hematological malignancies, Myeloid cells, Immune checkpoint, Targeted therapy, Targeting myeloid cells

## Abstract

Hematological malignancies are a diverse group of cancers that originate in the blood and bone marrow and are characterized by the abnormal proliferation and differentiation of hematopoietic cells. Myeloid blasts, which are derived from normal myeloid progenitors, play a central role in these diseases by disrupting hematopoiesis and driving disease progression. In addition, other myeloid cells, including tumor-associated macrophages and myeloid-derived suppressor cells, adapt dynamically to the tumor microenvironment, where they can promote immune evasion and resistance to treatment. This review explores the unique characteristics and pathogenic mechanisms of myeloid blasts, the immunosuppressive roles of myeloid cells, and their complex interactions within the TME. Furthermore, we highlight emerging therapeutic approaches targeting myeloid cells, focusing on strategies to reprogram their functions, inhibit their suppressive effects, or eliminate pathological populations altogether, as well as the latest preclinical and clinical trials advancing these approaches. By integrating insights from these studies, we aim to provide a comprehensive understanding of the roles of myeloid cells in hematological malignancies and their potential as therapeutic targets.

## Background

Hematological malignancies (HMs) refer to a set of pathological states characterized by stasis in differentiation and uncontrolled proliferation of immature blood cells known as blasts, hindering normal hematopoietic processes [1]. The incidence of HMs has been increasing for the past 30 years, reaching over 1.3 million cases globally in 2019, and is projected to reach 4.6 million in the year 2030, causing severe burdens on both the physical and financial well-being of the human race [2–4]. Although effective treatments are urgently needed, understanding the pathogenic mechanisms underlying heterogeneous HMs remains challenging. Of numerous mechanisms, two are involved throughout the entire process from pathogenic state to drug resistance development. Sequential gene mutations are the key initiators that drive the transition from clonal hematopoiesis to malignancies [5, 6]. By 2017, more than 70 mutated genes were reported to be associated with the pathogenesis of HMs through the modulation of various pathways, such as those involved in DNA repair or ribosomal abnormalities, to determine the genetic predisposition to the disease. [1] Another pivotal survival strategy of blasts is immune evasion [6, 7]. This balance is aggravatingly shifted in HMs, as malignant cells mostly convert from myeloid and lymphoid lineages, which are physiologically crucial responders in immune surveillance.

Myeloid cells are vital regulators that alter the aforementioned dysregulated immune response and modulate malignancy survival. [8] In the tumor microenvironment (TME), myeloid cells initiate dichotomous functions through polarization to distinct subtypes while receiving molecular cues released within the TME [8]. These cells are not stationary in light of their high plasticity to acquire different phenotypes, which are considered to be shaped by various signals in the TME, resulting in heterogeneous biological features [9]. To date, a certain number of surface cellular markers have been recognized to identify myeloid subpopulations and develop therapeutic targets. Myeloid cells, including tumor-associated macrophages (TAMs) and myeloid-derived suppressor cells (MDSCs), are considered pivotal in constructing the immune environment at the site of malignancies, with strategies that target these cells from multiple angles, from cellular development to immune functions. Myeloid-derived blasts in HMs are also discussed with respect to HMs, and their targeted therapy is also mentioned below.

Novel technologies, such as single-cell analysis and gene sequencing, have significantly improved the stratification management of HMs, which is moving toward precision medicine from diagnostic accuracy to prognostic monitoring [10]. With diversified advances in technological development, potential therapeutic approaches that target signaling pathways in myeloid cells are continuously emerging, especially in the treatment of solid tumors [8, 11]. However, targeting myeloid cells for hematological malignancies has not been discussed as much. This could be attributed to the shared expression of surface markers between malignant and normal myeloid cells, making the targeted therapy difficult to selectively eliminate the former without impairing the latter, which inevitably leads to immunosuppression and hematopoiesis inhibition. [12] Another underlying hurdle is that, unlike within solid tumor which often presents in a localized mass, blasts in HMs circulate throughout the body and construct supportive microenvironment in the bone marrow (BM), posing challenges to drug delivery, retention and effectiveness [13]. To address this issue, we present some recent studies that have targeted myeloid cells for the treatment of hematological malignancies.

## TAMs

TAMs originate from circulating monocytes and undergo functional polarization in response to various signals within the TME, including tumor-derived cytokines, metabolic cues such as lactate accumulation and hypoxia, all of which contribute to their immunosuppressive phenotype and support tumor progression. [14] Hence, macrophages observed in the TME often resemble an inhibitory M2 phenotype which serves as a negative prognostic marker in HMs, expressing and releasing immunosuppressive factors to promote immune evasion of malignant cells and impede immune clearance by T cells. [15, 16] It is reported that M2 macrophages can then be classified into subgroups M2a to M2d by their stimuli and immune functions, highlighting the heterogeneity of TAMs population [17]. Considering their high degree of plasticity in differentiation, targeting the M2 polarization of macrophages from different angles in the pathology of HMs, such as metabolic reprogramming, is becoming a feasible goal of macrophage immunotherapy. [14] Physiologically, macrophages are widely distributed in tissues, maintaining homeostasis and exerting immune defense through phagocytosis and secretion of cytokines using various sensors [18]. Targeting phagocytic immune checkpoints (ICs) or other important cellular events, such as recruitment, can also reinforce the ability of macrophages to eradicate malignant cells.

### Targeting macrophage polarization

Acute myeloid leukemia (AML) blasts can polarize leukemia-associated macrophages (LAMs) to the M2 phenotype, which further supports the survival of blasts by imposing phagocytic resistance and augmenting mitochondrial metabolism. [19] Liu et al. discovered that macrophage polarization affects AML cell growth in a murine model in vitro, and chenodeoxycholic acid may have contributed to this effect by metabolic reprogramming [20]. Blockade of T-cell immunoreceptor with Ig and ITIM domains (TIGIT), a coinhibitory factor initially found on T cells but also expressed on macrophages, can also reprogram M2-like LAMs into an M1-like phenotype when cocultured with AML cells in vitro, with increased secretion of M1 cytokines [21]. Macrophage receptor with a collagenous structure (MARCO) expressed on TAMs indicates an unfavorable prognosis among various malignancies, including lung cancer. [22] Anti-MARCO can repolarize M2 to M1 macrophages and restore the cytolytic activity of NK and T cells while turning down Treg cells, eventually blocking tumor growth and metastasis in lung cancer [23]. Among HMs, a higher level of MARCO is correlated with better OS in diffuse large B-cell lymphoma (DLBCL) but is negatively correlated with the tumor mutation burden and subsequent expression of neoantigens in AML. [22] This finding suggests less immune recognition and a lower chance of response to immunotherapy, which can potentially be reversed by MARCO blockade for further investigation. The distinct role of MARCO in various cancer types highlights the potential of studying macrophages in AML and other malignancies.

Other targets include interleukin-10 receptor (IL-10R), migration inhibitory factor (MIF), MER proto-oncogene tyrosine kinase (MerTK) and let-7b. Blockade of IL-10R with a monoclonal antibody (mAb) reduces M2 polarization of TAMs and suppresses the growth of multiple myeloma (MM) through the STAT3 pathway both in vitro and in vivo [24]. Spertini et al. reported that inhibiting macrophage MIF with granulocyte macrophage-colony stimulating factor (GM-CSF) affects the phenotypic reprogramming of macrophages from M2 to M1 in both in vitro and in vivo AML models, hence inducing the apoptosis of blasts and mitigating therapeutic resistance [25]. Cruz et al. reported that the inhibition of MerTK decreased the transformation of LAMs from M2 to M1 macrophages and increased the production of T-cell-activating cytokines via the activation of NF-κB [26]. Tian et al. reported an abnormal gene, let-7b, that contributed to promoting the conversion of LAMs to the M2 subtype, of which M1 repolarization via the Toll-like receptor and NF-κB pathways and inhibited AML development were inhibited [27].

### Targeting macrophage phagocytosis

The functional expression of phagocytic ICs is ubiquitous on myeloid cells, which transmit inhibitory signals upon binding to their ligands to suppress phagocytosis and mediate immune tolerance in physiological situations, whereas the expression of these ICs is induced by cancer cells, resulting in immune evasion [28]. Classic ICs, including cytotoxic T-lymphocyte antigen 4 (CTLA-4) and programmed cell death protein 1 (PD-1), which mostly affect T-cell activation, have been thoroughly characterized and developed into FDA-approved drugs with high efficiency for HMs [29]. Targeting phagocytic ICs is considered safer, as the cellular components are completely phagocytosed, with no segments remaining [28]. Phagocytic ICs, especially CD47 and other developing molecules, with relevant immune checkpoint inhibitors (ICIs) for HMs are mentioned below.

The cluster of differentiation 47 (CD47), which is expressed in the majority of human cells, is a highly glycosylated integrin-associated protein with a single extracellular V-set immunoglobulin superfamily domain that can bind with the NH2-terminal domain of signal regulatory protein α (SIRPα), which is observed in all myeloid cell types [30]. As the first identified myeloid-specific phagocytosis IC, CD47 was originally found on red blood cells (RBCs) and functions to regulate self-eradication by spleen macrophages [31]. CD47 was subsequently discovered to be a versatile molecule that initiates the regulation of major cellular events, including propagation and apoptosis, which are altered by abnormal genes during the pathogenesis of numerous human malignancies [28]. Elevated CD47 expression is observed among various HMs, including myeloid leukemia, non-Hodgkin lymphoma (NHL) and classical Hodgkin lymphoma (cHL), which exhibit protumor activities, predict adverse outcomes, and are emerging as effective targets [32–34]. While the majority of the functions of CD47 have been studied by binding to SIRPα on immune cells, it has been revealed that CD47 mediates prosurvival signals by interacting with thrombospondin-1 on malignant cells in cutaneous T-cell lymphoma (CTCL) [35].

CD47 blockade can also stimulate macrophage cytokine secretion, induce natural killer (NK) cell-mediated antibody-dependent cell-mediated cytotoxicity (ADCC), and promote dendritic cell-mediated antigen presentation to activate the adaptive immune system [28]. The inhibition of CD47/SIRPα binding eventually leads to the apoptosis of blasts, which is a promising strategy for myeloid immunotherapy and a major field of ICIs in the treatment of HMs. As elaborately organized by Liu et al., more than 20 clinical trials targeting CD47 are in ongoing trials, mostly in phase I/II. [28] In a phase Ib study of magrolimab, which is the first CD47 mAb with rituximab in relapsed/refractory (R/R) NHL, 50% of 22 patients achieved an overall response (OR), with 36% achieving a complete response (CR), and 90% remaining among those who responded at the 6-month mark [36]. The results of a 3-year follow-up of a phase Ib/II clinical trial in R/R indolent NHL revealed that after administration, the overall response rate (ORR) reached 52.2%, with 30.4% CR, while remaining fairly tolerable [37]. As reported recently, of 33 R/R DLBCL patients, cocktail therapies combining first-in-human CD47 antibody magrolimab, rituximab plus gemcitabine-oxaliplatin achieved an ORR of 52%, with 39% achieving CR, maintaining a 66.6% 12-month response rate, but with a grade 3 anemia rate of 60.6%. [38]

Finding synergistic targets to initiate combined blockade not only increases efficiency but also ensures safety, hence continues to be the primary strategy. Multiple ICs are expressed on blasts, dual IC targets can provide complementary effects. Wang et al. designed a CD47-programmed death-ligand 1 (PD-L1) bispecific antibody (BsAb) with high PD-L1 affinity and low CD47 affinity [39]. This enables binding precedence over PD-L1, which is more specific for blasts than for RBCs, while providing unique immune activation, which functions mainly through the CD47/SIRPα pathway. This dual targeting approach has also been revealed to have potential synergistic effects on macrophages in CTCL cells not only by enhancing phagocytic activity but also by directly strangling CTCL cell growth through cell death-related pathways [40]. Brauneck et al. reported that blockade of TIGIT on macrophages can significantly augment the anti-CD47-mediated phagocytosis of AML cells in vitro. [21] It has also been reported that CD47-leukocyte immunoglobulin-like receptor B1 (LILRB1) is a promising dual target in lymphatic malignancies, as enhanced antibody-dependent cell-mediated phagocytosis (ADCP) by macrophages was observed when these cells were cocultured with DLBCL and Burkitt cell lines [41]. High levels of sialic acid-binding immunoglobulin-like lectin-10 (Siglec-10) and SIRPα expression on macrophages result in increased phagocytosis of the mantle cell lymphoma (MCL) cell line in response to CD24-CD47 mAbs to a greater degree than to a single mAb, especially when rituximab is used simultaneously [42]. In addition to targeting functional surface molecules, dual targets of CD47 can also include cell markers for recognition. In addition to the aforementioned anti-CD20 agent, the administration of the CD19-CD47 BsAb also enhanced TAM-mediated phagocytosis of blasts in a mouse xenograft B-cell lymphoma model, and the combination of CD19 and CD47 blockade was highly efficient in B-ALL mouse models [43, 44]. While biblockades of surface markers and CD47 have synergistic effects on several HMs, the myeloid marker CD33 is rarely observed in BsAbs because of the heterogeneity of AML blasts. Further development requires a better understanding of molecular signaling networks, especially when the cotarget effects are ambiguous [45].

Modification of novel epitopes of CD47 antibodies is also an approach. Lemzoparlimab, a novel antibody with a unique N-linked glycosylation site, appears to show superior specificity for CD47 as a result of glycosylation near the binding epitope of CD47 on RBCs [46]. In a phase I trial of lemzoparlimab in R/R AML, no dose-dependent hematological adverse effects (AEs) were reported at a weekly dose of 10 mg/kg. [47] Lemzoparlimab also demonstrated high effectiveness, as seen in a phase IIa trial of newly diagnosed (ND) HR MDS, of 28 patients who received more than 3 cycles of lemzoparlimab combined with azacitidine, 23 responded to treatment with elevated and sustained prophagocytotic signals in blasts [48].

Among the unparalleled concerns, the primary treatment-emergent adverse effects (TEAEs) of blocking CD47 are mostly hematological toxicities, including anemia, which occurs at a notable frequency of approximately 50% and a degree of above grade 3 TEAEs, as seen in the results of several clinical trials, even with priming doses [36, 49, 50].

Reduced hematological AEs can be achieved by the SIRPα fusion protein with an inactive Fc region, although it may reduce the efficacy [51]. In a phase I trial of a SIRPα-IgG1 Fc in R/R HMs, TEAEs were anemia at 13% and thrombocytopenia at 26%, while the ORR was merely 13% [52]. Amelioration can be achieved by a bispecific fusion protein with a functional Fc domain. IMM0306, a fusion protein of anti-CD20 IgG1 with a SIRPα domain, shows high affinity for CD47-positive T-cell leukemia cell lines and more intensified ADCP and complement-dependent cytotoxicity (CDC) than does rituximab alone, with a low level of RBC binding [53]. Although reported to be well tolerated, 10 out of 11 R/R indolent NHL patients who received IMM0306 with lenalidomide experienced TEAEs above grade 3 [54]. A CD123 and SIRPα fusion protein markedly eliminated AML blasts and LSCs via enhanced phagocytosis, which is superior to the use of high-affinity CD47-CD123 antibodies in vitro [55]. The balance between the efficacy and safety of fusion proteins, in which structural amendments may address a change, should be further tested.

Other strategies include the use of smaller materials and novel delivery methods. Hu et al. designed a chimeric peptide of modified CD47/SIRPα and PD-1/PD-L1 blocking peptides, the delicate size of which provides minor immunogenicity [56]. Luo et al. constructed mesoporous silica nanoparticles with an anti-CD47 antibody on the surface and doxorubicin, initiating synergistic phagocytosis enhancement of macrophages with targeting effects in four solid tumor cell lines [57]. Its effect on HMs adjusted with suitable chemotherapeutic drugs should be further investigated.

In addition to CD47, novel phagocytic targets are emerging, with promising preclinical results. CD24, which was first identified as a heat-stable antigen, has gradually become known to be a heavily glycosylated membrane protein with multiple N/O-glycosylation sites and is highly variable among human cells [58]. CD24 is expressed in many human cells, preferably in cells with high proliferation potential, and appears to decline as cells develop, as is the case for multiple stem cells, as well as B-cell and T-cell progenitor cells, but not fully mature cells [59]. Various physiological functions of CD24 include regulating the clonal expansion and homeostatic proliferation of T cells [60]. Dysfunction of this immunosuppressive feature contributes to the pathogenesis of a number of cancers and autoimmune diseases. In cancer immunology, CD24 is overexpressed on malignant cells such as ovarian and breast cancer cells, signaling that 'don’t eat me' through its sialylated glycans by binding with the amino-terminal V-set extracellular domain of Siglec-10, mainly on TAMs, to negatively affect phagocytosis [60].

In various HMs, CD24 expression is broadly elevated, and CD24 blockade has shown enhanced phagocytic effects in multiple cell experiments and xenograft models. Compared with CD47, the absence of CD24 on human RBCs can prevent off-target toxic anemia, suggesting a promising perspective for targeted treatment [60]. The number of macrophages in CD24-positive DLBCL blasts and the intensity of phagocytosis in MCL blasts are markedly decreased in vitro, with the latter presenting superior specificity and effectiveness in comparison with CD47 blockade [61, 62]. Currently, clinical trials targeting CD24 with HMs include mAbs ALB9 and BL13 in the treatment of B-lymphoproliferative disorders, which are highly tolerable, and efprezimodα in the treatment of graft-versus-host disease (GVHD) in AML patients with hematopoietic stem cell transplantation (HSCT), which was terminated with nonideal results [63]. Selecting eligible contexts to conduct further experiments is the key point for exploring CD24 blockade.

The results also show that the enhanced phagocytosis induced by CD24 blockade is mediated through the Siglec-10 inhibitory pathway, not by Fc-mediated opsonization, as investigated in solid tumors. Shen et al. identified a hydrolysis-resistant dual-targeting blocking peptide for CD24/Siglec-10 and PD-1/PD-L1, which induced phagocytosis and achieved reduced tumor growth with a combination of radiotherapy in several cancer cell lines and tumor models [64]. The latter finding conflicts with B-cell ALL, in which blasts with low CD24 expression indicate intrinsic radiation resistance [65]. This phenomenon needs further investigation, as the anti-CD24 strategy applied in HMs is rather less common than it is in solid tumors.

However, within HMs, the expression of CD24 seems to be inconclusive. The function and prognostic value of CD24 in MM seem paradoxical, as previously reported [66, 67]. Contradictory results have also been reported with respect to DLBCL, where CD24 levels indicate an unfavorable prognosis in three independent datasets related to MYC aberrations, whereas interestingly, CD24 levels are not correlated with OS or mAb treatment efficacy [61, 62]. For myeloid malignancies, although CD24 is said to be a highly specific marker of M4/M5 leukemia, [68] Barkal et al. reported low expression of CD24 in AML, possibly due to the distinct phenotypes of AML blasts [60].

The use of phagocytic ICIs is considered one of the major strategies of targeting phagocytosis. To improve therapeutic effectiveness while maintaining tolerable toxicity, finding combined targets, modifying specific binding epitopes and developing novel drug structures and delivery methods might be useful in the future [51]. On the other hand, the function of novel molecules which firstly emerged as T-cell ICs, especially LILRB1 or PD-1, in phagocytosis for HMs along with their further application should continuously be researched [69].

### Targeting macrophage recruitment

Colony stimulating factor 1 (CSF-1), which physiologically regulates the proliferation, differentiation and recruitment of macrophages via CSF-1 receptor (CSF-1R), plays an important role in the acquisition of cellular malignant characteristics, including metastasis, by regulating the function of TAMs [70]. Among HMs, CSF-1R activation in T-cell lymphomas reportedly promotes tumor growth via the AKT/mTOR pathway [71]. CSF-1-targeted therapy might be a promising strategy for treating HMs. In a phase I/II study of the selective CSF-1R inhibitor JNJ-40346527 in R/R cHL, 90.5% of treated patients experienced at least 1 AE, 37% of whom were above grade 3, whereas 55% reached stable disease (SD) and 40% had progressive disease (PD) during treatment [72]. In a follicular lymphoma (FL) mouse model, CSF-1R and the secretion of CSF-1 are both seen elevated, with the latter promoting M2 polarization in the TME, which can be reversed by CSF-1R inhibition prior to M1, resulting in reduced tumor burden in vivo [73]. However, the suitability of CSF-1R inhibitors for the treatment of AML is debatable because of concerns about the impairment of the weakened immune response, as CSF-1R inhibition can cause long-lasting damage to circulating macrophages [74]. Selectivity of the CSF-1R inhibitor for M1/M2 macrophages seems to be the key determinant of whether its effects are protumor or antitumor.

### CAR-M

**Fig. 1:**
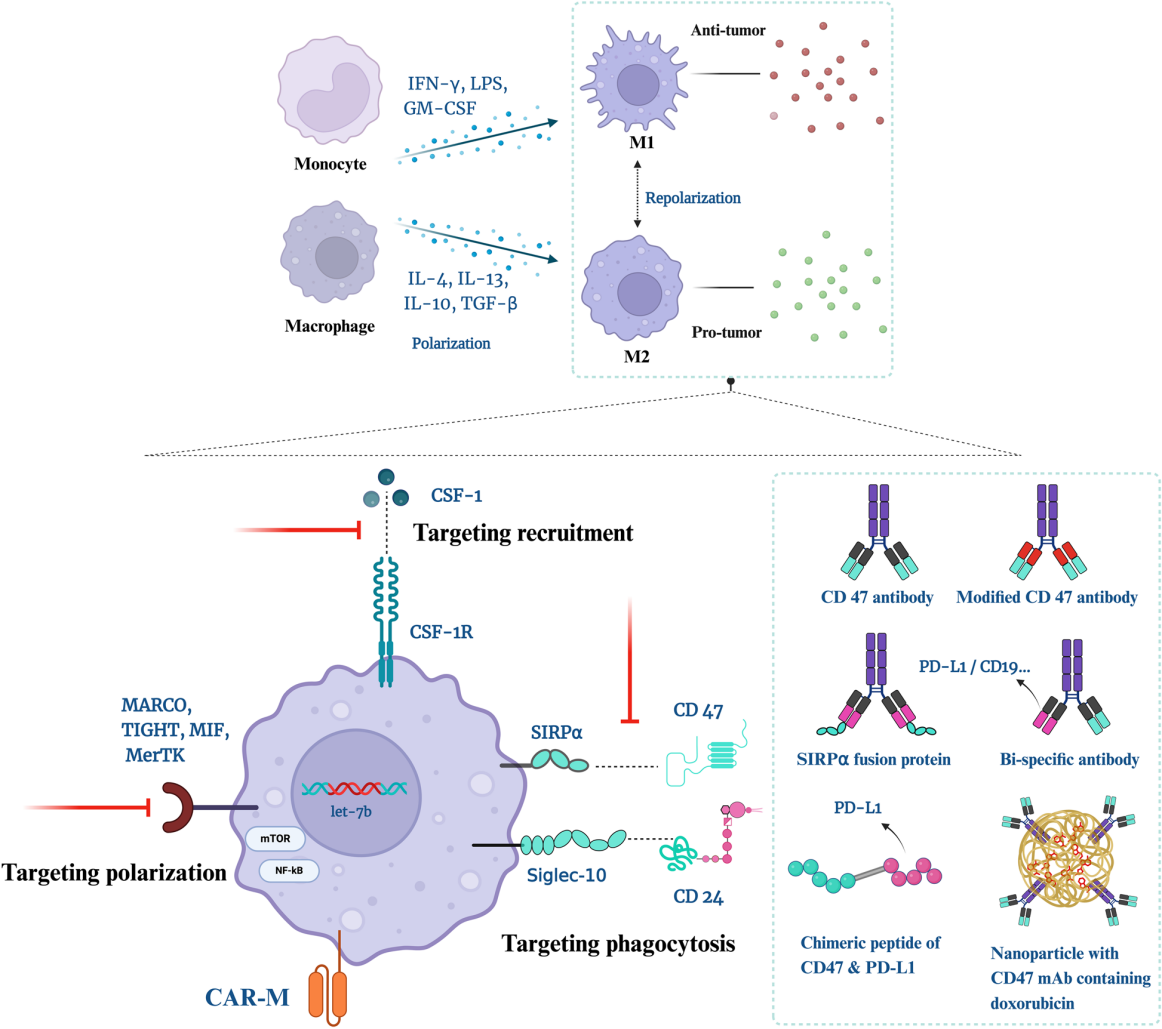
Targeting TAMs for HMs. Monocyte and macrophage in the TME are polarized into anti-tumor M1 and pro-tumor M2. They can repolarize under molecular signals due to their high plasticity. Strategies targeting these TAMs include promoting M2 polarization, suppressing phagocytosis and inhibiting recruitment. CAR-M is another perspective strategy. Of all therapeutic approaches, targeting phagocytic ICs majoring CD47 is at the forefront of development. Traditional strategies targeting CD47 include monoclonal antibody, modified antibody, SIRPα fusion protein, bi-specific antibody. Novel targeted strategies are explored using chimeric peptide and nanoparticle

Chimeric antigen receptor-macrophage (CAR-M) is a novel technological field focused mainly on treating solid tumors and contributes to its ability to infiltrate and phagocytose tumor cells in the TME, whereas CAR-T cells have made tremendous progress in the past decade with respect to the treatment of HMs [75]. CY-M11, the first human CAR-M in patients with ovarian cancer and malignant peritoneal mesothelioma, is generated from peripheral blood mononuclear cells (PBMCs) with mRNA encoding an anti-mesothelin CAR constructed in less than 24 h [76]. It has shown great tolerability and feasibility, with 4 out of 11 patients receiving and sustaining SD within months after administration, with most reported AEs being grade 1/2. As a newly emerging targeted therapy, CAR-M cells are anticipated for the treatment of HMs. Zhang et al. designed an anti-CD19 CAR-M with multiple types of stem cells, each of which exhibited enhanced and antigen-dependent phagocytosis of CD19 + ALL cells, increased proinflammatory responses and enhanced phagocytic capacity in vitro and antitumor effects in vivo [77]. In another study, Abdin SM et al. introduced the technology of human induced pluripotent stem cells (iPSCs)-derived CAR-M in which macrophages showed enhanced ADCP toward CD19^+^ leukemia cells in vitro, while being repolarized to a M1-like state [78]. With respect to CAR-M clinical trials, few studies are currently in progress, mostly targeting mesothelin and human epidermal growth factor receptor 2 (HER-2) in solid tumors, and only one attempt to treat HMs has been tested clinically [79]. This phase I/II study focused on the tolerability and efficacy of a CD5-targeted CAR-M called MTX-TCL-101 in patients with R/R peripheral T-cell lymphoma (PTCL) who were receiving multiple ascending doses. The drug had received a fast track designation from the FDA, and patients did not show signs of cytokine release syndrome (CRS) or suffer from cytotoxicity after the first administration [80]. To date, no results have been published, and although the estimated primary completion is due to 2024–11, the study has been suspended for temporary operational reasons [81]. For future research, studies should focus on manufacturing and indications. The summary of strategies for HMs targeting macrophages is seen in Fig. 1.

## MDSCs

MDSCs are a group of heterogeneous immunosuppressive myeloid cells generated in the context of pathological status with certain immature biomarkers, resulting in a deficiency in further differentiation [82]. While it is debatable whether MDSCs are a distinct myeloid lineage or a pathologically functional state, MDSCs are generally classified by their derivation into two subtypes: monocytic MDSCs (M-MDSCs), written as CD11b^+^CD14^+^CD33^+^HLA-DR^−^ and granulocyte-like MDSCs/polymorphonuclear MDSCs (G-MDSCs) with CD11b^+^CD15^+^HLA-DR^−^, which commonly function in the inhibition of T and NK cell activation [45]. In human malignancies, which are shaped by molecules released by adjacent cancerous cells, MDSCs play indispensable roles in tumorigenesis, metastasis, and secondary therapeutic resistance and act as targets for multiple nonspecific existing agents [83].

### MDSCs in HMs

Within HMs, the abovementioned pattern exists with MDSCs representing early relapse, multidrug resistance, and a worse prognosis [84, 85]. While G-MDSCs are considered unfavorable in most cases, a recent study revealed that G-MDSCs in peripheral blood (PB) are related to better outcomes and less relapse in refractory cHL, whereas M-MDSCs adjacent to Hodgkin and Reed–Sternberg (HRS) cells are associated with a worse prognosis [86]. In ND DLBCL adults, high levels of surface triggering receptor expressed on myeloid cells 2 (TREM2) on circulating M-MDSCs are poor prognostic factors for both OS and progression-free survival [87]. The trajectory of the development of this target paves the way for understanding the complexity of heterogeneous MDSCs by narrowing the specific expression of different MDSC subtypes and initiating interventions. Further investigations can be initiated using an inhibitory receptor antibody for treating malignancies.

### Inhibition of MDSC formation

Identifying the driving force of MDSCs helps in understanding the reversal of these cells in immunotherapy. Chemotherapy has been shown to inhibit the development of MDSCs. 5-Fluorouracil can suppress the early stage of expansion of MDSCs in a lymphoma mouse model [88]. AML-derived extracellular vesicle uptake induces the formation of MDSCs via surface protein palmitoylation, which is a potential target for immune enhancement [89]. Peng et al. discovered that elevated S100 calcium-binding protein A4 (S100A4) mediates the expansion and function of MDSCs via the GP130/JAK2/STAT3 signaling pathway in AML blasts, which restores T-cell activation via S100A4 silencing. [90]

### Interfering with the function of MDSCs

Blocking certain pathways to impair the function of MDSCs and regain immunocompetence is also a feasible approach for targeting HMs. However, this is hindered because the targets exhibited by MDSCs are nonspecific, which makes it difficult to evaluate treatment efficiency. Synergistic effects were observed following MDSC inhibition. Daneshmandi et al. reported that β2-adrenergic receptor activation enhances MDSC function through metabolic reprogramming of mitochondria and that the combination of doxorubicin and propranolol can improve survival in C1498 AML and EL4 lymphoma models [84]. The release of tumor necrosis factor α (TNFα) by AML blasts can be induced by cytarabine, which promotes MDSC expansion and function via the IL-6/STAT3 and NF-κB pathways, which can be impaired by TNFα blockade, providing a novel therapeutic strategy [91]. A phase II clinical trial of an inhibitor of indoleamine 2,3-dioxygenase (IDO) in MDS patients who previously received azacitidine reported SD in 80% and PD in 20%, with fair tolerance [92].

### Targeting MDSC recruitment

Circumstantial evidence of targeting MDSC recruitment in HMs has emerged. Mesenchymal stem cells in the TME contribute to chemoresistance in AML, which is initiated by increased IL-6 secretion through JAK2/STAT3 signaling, resulting in positive feedback [93]. IL-6 inhibitors can potentially interfere with this pathway while decreasing MDSC recruitment. Matrix metalloproteinase 8 (MMP8) expression independently indicates a poor prognosis by regulating the recruitment of G-MDSCs in FGFR1-mutated leukemia blasts, which can be reversed by inhibition, resulting in significantly improved survival in vivo with impaired G-MDSC recruitment [94]. M-MDSCs recruitment to the TME is mainly mediated by chemokine ligand 2 (CCL2) /chemokine receptor type 2 (CCR2) axis, and inhibitors of this axis have potential therapeutic effects in various cancers [83]. CCL2 is also reported to drive resistance to trametinib in AML blasts, while inhibition of CCR2 can reinstate the sensitivity to trametinib [95]. Though there is not direct suggestion, whether MDSCs are involved in this mechanism may require further investigation. IL-6 inhibitors can potentially interfere with this pathway while decreasing MDSC recruitment.

### Novel prospects

**Fig. 2:**
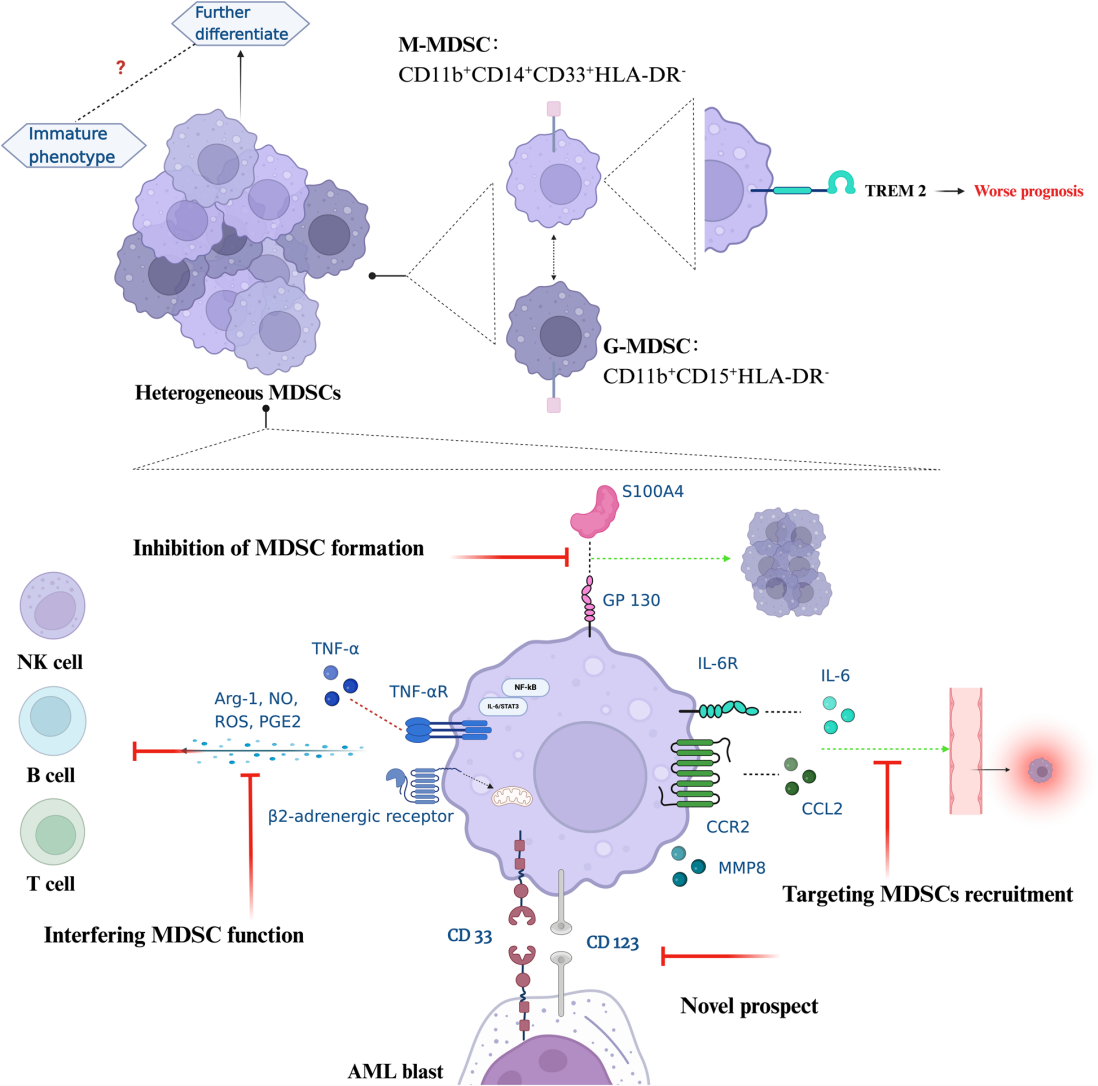
Targeting MDSCs for HMs. MDSCs exhibit a heterogeneous population, including M-MDSC and G-MDSC subtypes, with an immature phenotype possibly capable of further differentiation. Elevated expression of TREM2 on M-MDSCs correlates with poor prognosis. Strategies targeting these MDSCs include inhibition of MDSC formation, interfering with MDSC function and targeting MDSC recruitment. Novel therapeutic approaches also target specific markers, such as CD33 and CD123, expressed on MDSCs and AML blasts

The expression of common antigens, specifically CD33, on immature M-MDSCs and AML blasts in targeted therapy may have favorable effects and distinct functions. An in vivo experiment revealed that the effectiveness of a CD33-CD3 BsAb in eradicating AML blasts is enhanced by M-MDSC inhibition by targeting CD33 [96]. The level of MDSCs is a predictor of responsiveness to therapy. CD123-NKG2DL dual-targeted CAR-T cells can selectively weaken M-MDSCs while killing AML blasts, as the two targeted antigens are expressed on both AML blasts and M-MDSCs [97]. This result suggests the possibility of extended remission duration by cotargeting effects on MDSCs. A CD33-CD3 BsAb trial conducted in R/R AML and MDS revealed no hematological responses, whereas 94.1% of patients experienced at least 1 above grade 3 TEAE [98]. Only transient reductions were observed in a few patients, suggesting that compensatory mechanisms in the human immune environment might have occurred, which needs to be further investigated before clinical use. The summary of strategies for HMs targeting MDSCs is presented in Fig. 2.

## Myeloid-derived blasts in HMs

In myeloid malignancies, normal myeloid cells at different stages become blasts that have sustained proliferative ability and impaired immune function in the context of a variety of mutations. Philadelphia chromosome-positive chronic myeloid leukemia (CML) and AML are the focus of discussion, with the former originating from a single chromosome translocation and the latter resulting from the accumulation of multiple genetic events [99].

### Brief on CML treatment

Contributing to the discovery of the pathogenic BCR-ABL fusion gene and its targeted therapy, treatment for CML is rather favorable using effective multi-generation tyrosine kinase inhibitor (TKI). Single oral imatinib achieved an estimated 10-year OS of 91.1% among those with a molecular response at 12 months and 85.3% among those without, while showing minimum AEs [100]. The challenge in the use of TKIs is drug-induced mutation and subsequent resistance. The combination of ponatinib and asciminib achieved a complete hematologic response in a patient who developed T315I/E355G mutations, as asciminib reversed the sensitivity of the BCR-ABL1 complex to ponatinib, which represents a feasible strategy for TKI therapy [101]. Despite few cases, CML patients present a favorable prognosis with rare drug resistance, good therapeutic tolerance and a high treatment-free remission rate [102].

### Therapeutic challenges and strategies of AML

Compared with CML, LSCs in AML present greater diversity and complexity in terms of their immunophenotype, genetics and clonal evolution [99]. Standard favorable frontline treatment for AML includes induction chemotherapy followed by HSCT, harvesting a 5-year OS of less than 30% and 10% for R/R patients [103]. In contrast to the progress made in some lymphoid malignancies and CML with known sweet spots and relevant agents showing long-lasting effectiveness, the development of targeted treatment for AML has more obstacles [104]. The acquisition of practicable targets constantly falls short of phenotypical heterogeneity and dynamic changes through pathological development, ultimately resulting in multiresistance to existing approaches [105]. Therefore, AML is characterized by rapid progression, a high recurrence rate, and an unfavorable prognosis; thus, novel treatments are urgently needed [103]. With respect to AML, targeting mutated genes and surface biomarkers of blasts are primary therapeutic goals.

### Targeted therapy for mutated genes

Gene mutations, such as FMS-related tyrosine kinase 3 (FLT3) and isocitrate dehydrogenase (IDH), frequently occur in AML, initiating pathogenesis and acting as potential therapeutic targets [1]. FLT3 is a receptor tyrosine kinase expressed on blood cells at an early stage that promotes cell proliferation and differentiation [106]. In AML, the FLT3-activating mutation constitutively activates FLT3 kinase and promotes the survival of blasts, especially with FLT-3 internal tandem duplication (ITD) being an adverse prognostic mutation [107]. FLT3 inhibitors (FLT3i) reverse the activation of kinases through binding with the intracellular domain and suppressing downstream prosurvival signaling [106]. The first FLT3i, midostaurin, received FDA approval in 2017, as it prolonged OS and event-free survival when added to chemotherapy in all FLT3-mutated AML subgroups [108]. Followed by single FLT3i gilteritinib with an estimated 2-year OS of 20.6% compared to 14.2% with salvage chemotherapy, being a suboptimal alternative for non-first-line treatment [109, 110]. Recently, FDA-approved FLT3i has shown promising prospects, as in a randomized phase III trial of 539 patients with FLT3-ITD-positive AML who were treated with quizartinib and placebo, the median OS was 32 months and 15 months, respectively [111]. The preclinical development of FLT3i is continuously emerging. An Fc-optimized FLT3 antibody with an improved CD16 affinity Fc region had an OR of 46% and moderate AEs in a phase I trial in persisting/increasing minimal residual disease (MRD) AML patients [112]. Ansari et al. manufactured a lipopolymer with FLT3 siRNA that showed enhanced cytotoxic activity and selectivity toward FLT3-ITD-mutated cells when treated with daunorubicin and gilteritinib [113]. Another significant mutation of AML is IDH, where mutated IDH produces pro-tumor metabolin D-2-hydroxyglutarate, altering epigenetic traits and metabolic process by suppressing differentiation [114]. Though it remains contradictory whether IDH mutation is a prognostic factor for AML, inhibitors of IDH show considerable therapeutic effects by reducing the oncometabolite. Ivosidenib and enasidenib, for example, working as small molecule inhibitors of IDH1 and IDH2 enzymes, are showing fair safety and efficacy in IDH mutated AML [115, 116]. TP53 is a crucial tumor suppressor gene regulating cell cycle, and its mutation is markedly associated with complex karyotypes and poor prognosis in myeloid malignancies [117]. Eprenetapopt, a p53 reactivator is also a promising therapeutic strategy, as it has an ORR of 64% with 38% being CR with venetoclax and azacitidine in TP53 mutated AML in a phase I study [118]. Approaches targeting IDH1/2 and TP53, along with other selected targeted therapies, are listed in Table 1.

**Table 1:** Myeloid cells and their targets for clinical application

Target cell	Target	Drug	Type	Effecacy/Clinical effect	Reference
**TAMs**	CD47	Magrolimab	mAb	ORR 52.2% (30.4% CR) in R/R NHL with rituximab	[37]
				ORR 24% (12% CR) with rituximab, ORR 52% (39% CR) with rituximab and gemcitabine-oxaliplatin in R/R DLBCL	[38]
				ORR 75% (33% CR, 40% CR in TP53 mutated ones) with azacitidine in HR-MDS	[119]
		Lemzoparlimab	mAb	1 morphologic leukemia-free state in 5 R/R AML	[47]
				7 ORR of 8 (3 CR) in R/R NHL with rituximab	[120]
				ORR 82.1% (2 CR, being TP53 mutated ones) with azacitidine in ND HR-MDS	[48]
		Gentulizumab	mAb	Increased phagocytosis for B-cell malignant cell lines and decreased migration of AML cell lines in vitro	[121]
		Ligufalimab	mAb	Significant antitumor effects of B-cell lymphoma cell lines in vivo and enhanced phagocytosis of multiple T/B-cell malignant cell lines in vitro	[122]
		SIRPα‐αCD123 fusion protein	fusion protein	Enhanced AML cell phagocytosis in vitro and specificity in eradication of LSCs in vivo	[55]
		IMM0306	fusion protein	Increased ADCP, ADCC, CDC for T-cell leukemia cell lines in vitro and outstanding tumor growth inhibition of B-cell malignant graft models	[53]
		CD47/CD19 BsAb (NI-1701)	bispecific antibody	Increased TAM-mediated phagocytosis of B-cell lymphoma xenograft models	[43]
	CD24	CD24 antibody	mAb	Increased phagocytosis for MCL/CLL cell lines with CD47 mAb in vitro	[42]
**MDSCs**	IDO	INCB024360	small molecule inhibitor	Mean MDSC % 29.5% at baseline and 27.6% after treatment in MDS	[92]
	CCR2	RS504393	small molecule inhibitor	Enhanced sensitivity to MEK inhibitor and decreased proliferation and enhancing apoptosis of AML cell lines	[95]
	S100A4	siS100A4	si RNA	Downregulated MDSC expansion, secretion and impaired MDSC-mediated inhibition of T-cell activation in AML cell lines	[90]
**Myeloid-derived blasts**	CD33	Gemtuzumab ozogamicin	antibody‒drug conjugate	Improved 5-year OS by reducing relapse rate in patients with favorable cytogenetics and intermediate risk	[123]
	CD123	Pivekimab	antibody‒drug conjugate	ORR 21% (17%CR) with azacitidine and venetoclax in R/R AML	[124]
	E-selectin	Uproleselan	glycomimetics	35% CR with chemotherapy in R/R AML; 52% CR in elder patients with ND AML	[125]
				Increased OS of AML mouse with 5-azacitidine	[126]
	FLT3	Midostaurin	small molecule inhibitor	Prolonged OS (hazard ratio for death 0.78) and event-free survival with standard chemotherapy in FLT3 mutated AML	[108]
		Gilteritinib	small molecule inhibitor	Prolonged OS (hazard ratio for death 0.665) with standard chemotherapy in FLT3 mutated AML	[109]
		Quizartinib	small molecule inhibitor	Prolonged OS ((hazard ratio for death 0.78) with standard chemotherapy in FLT3 mutated AML	[111]
	IDH2	Enasidenib	small molecule inhibitor	ORR 40.3%(19.3% CR) with chemotherapy in IDH2 mutated R/R AML	[116]
	IDH1	Ivosidenib	small molecule inhibitor	Prolonged OS (24.0 vs. 7.9 months; hazard ratio for death 0.44) with azacitidine in IDH1 mutated ND AML	[115]
		Olutasidenib	small molecule inhibitor	ORR 41% and 46% with azacitidine in IDH1 mutated R/R AML; ORR 25% and 77% with azacitidine in IDH1 mutated ND AML	[127]
	P53	Eprenetapopt	small molecule reactivator	ORR of 64% (38% CR) with venetoclax and azacytidine in TP53 mutated AML	[118]
	BCR-ABL	Ponatinib	small molecule inhibitor	1-year OS of 81.3% and 2-year OS of 63.9% after HSCT in FLT3-ITD mutated AML	[128]
		Olverembatinib	small molecule inhibitor	Major cytogenetic response/complete cytogenetic response/major molecular response being 79.0%/69.0%/56.0% in CML chronic phase, 47.4%/47.4%/44.7% in CML accelerated phase	[129]
	BCL-2	Venetoclax	small molecule inhibitor	MRD-negative CR 86% vs. 61% with intensive chemotherapy. Improved event free survival, not OS	[130]
				Improved remission rate and OS (8.4 vs. 4.1 months) with low dose cytarabine in unfit ND AML	[131]

### Targeting CD33

CD33, a glycosylated transmembrane protein also known as Siglec-3 with a V-set extracellular domain that binds sialic acid, is a marker of myeloid progenitor cells including blasts, which regulates proliferation and differentiation [132]. As it is expressed in approximately 90% of AML blasts but not in nonhematopoietic tissues, CD33 is a great target for AML treatment. The first and only FDA-approved targeted antibody‒drug conjugate (ADC) is gemtuzumab ozogamicin (GO), which comprises a CD33 mAb and calicheamicin [133]. In a 280-patient phase III trial in AML, the CR rate was 81% with the addition of GO in standard treatment, whereas it was 75% in the control group, with a greater occurrence of hematological toxicity [133]. While it was once questioned for fatal AEs such as veno-occlusive disease and sinusoidal obstruction syndrome, GO has gradually returned to stage because of its high efficacy, with amendatory prevention and management [134]. Current research focuses mainly on the dosing amount and frequency, especially how to integrate a lower dose of GO safely into other intensive treatments [135]. In a recently conducted phase I trial in ND AML, specifically FLT3-mutated AML and core-binding factor leukemia, GO, along with midostaurin and intensive chemotherapy, was shown to be safe, with 75% achieving CR [136]. GO has been granted permission to be used as monotherapy for patients older than 2 years and in combination with intensive chemotherapy for patients at favorable risk [137]. Despite the bumpy road of development and the challenges in ensuring safety, GO still seems to be indispensable and holds considerable potential for CD33 + AML treatment.

CD33 CAR-T is a promising and relatively mature area of targeted therapy as it detects antigens independent of HLA [138]. In a phase I/II trial of CD33 CAR-T cells in R/R AML, 68% presented with CRS, with 21% being above grade 3 within the first 24 h after infusion, and CR was achieved in only 2 of 24 subjects [139]. The heterogeneity of AML blasts often renders single-targeting CAR-T cells ineffective, as they fail to achieve complete eradication for not recognizing all AML subpopulations including LSCs. To address this challenge, bispecific CAR-T cells are being developed to enhance specificity and reduce immune escape [105]. In a phase I trial of C-type lectin-like molecule-1 (CLL1)-CD33 combined with CAR-T cells, patients with FLT3-ITD-mutated AML achieved CR, with MRD-free individuals in the BM within 3 weeks after administration, and abundant CAR-T cells successfully expanded in both the PB and BM [140]. The manufacture of CD33-TIM3 CAR-T cells is also in progress, with the potential to eradicate LSCs with distinctive expression of TIM3 [141]. Another hurdle is the pan-expression of CD33 on AML blasts and normal hematopoietic progenitor cells, raising concerns about on-target, off-tumor toxicity. Kim et al. revealed that gene knockdown of CD33 provides additional specificity for CD33 CAR-T cells to eradicate AML blasts without damaging normal hematopoiesis because CD33 is not necessary for myeloid development and function [12]. Appelbaum et al. designed a dimerizing agent–regulated immunoreceptor complex for CD33 CAR-T cells in AML, which demonstrated rapamycin-dependent cytotoxicity to avoid off-target AEs while maintaining clinical efficacy [142]. Compared with the development of other HMs, developing CAR-T therapy for AML is especially challenging because of these challenges, hence making the selection of eligible target antigens critical [143].

CAR-NK cells exhibit a safer profile, as they have been shown to be tolerable in several clinical trials, which have been conducted mostly with HMs, providing novel insight for targeted therapy [144]. As NK cells can be applied allogeneically, CAR-NK cells constitute a practicable alternative for T-cell treatment due to the simplicity in manufacture. Automated-produced CD33 CAR-NK cells produced by Albinger et al. are reported to have killing effects that are indistinguishable from those of small-scale-produced CAR-NK cells both in vitro and in vivo, which provides a promising strategy for volume production [145]. Bexte et al. combined CD33 CAR-NK with CRISPR/Cas9, in which the removal of the natural killer cell receptor group 2 member a (NKG2A) functions synergistically in vitro and in vivo compared with a single approach [146].

### Targeting PSGL-1/E-selectin

Endothelial adhesion molecule 1 (E-selectin) is a type of calcium-dependent lectin found on endothelial cells that mediates endothelial-leukocyte adhesion, lymphocyte homing, and leukocyte rolling, mostly via binding to proteins or lipids with sialyl Lewis X/A [147]. The expression of E-selectin on the endothelium is constitutive only in BM and skin, whereas in other regions only when activated, enhancing the proliferation of human stem cells [147]. AML blasts release inflammatory cytokines to upregulate E-selectin expression in endothelial cells while increasing the expression of the E-selectin ligand fucosylation on their own surface [148]. Enhanced binding form a vascular niche to support the survival of blasts instead of normal hematopoietic stem cells (HSCs), which is also known as niche hijack. Furthermore, increased E-selectin interaction with P-selectin glycoprotein ligand-1 (PSGL-1) strengthens prosurvival signaling through the AKT/NF-κB pathways, contributing to MRD development and drug resistance, which also results in a protective sanctuary leading to subsequent relapse episodes.

Notably, E-selectin antagonists can protect normal HSCs and relieve BM suppression by inducing quiescence, while disrupting vascular niche-mediated chemoresistance in AML blasts, indicating that combining chemotherapy with blockade of E-selectin may be a promising therapeutic approach [148]. Moreover, monotherapy with a small-molecule antagonist of E-selectin uproleselan alone has a very limited effect on various AML cell lines, as shown in the article, whereas the combination of uproleselan with daunorubicin/cytarabine significantly improved survival in these cell lines [149]. Supplementary functions for both uproleselan and chemotherapy in terms of therapeutic sensitivity and erased AEs suggest a strategy for future investment in the compatibility of uproleselan with other therapeutic approaches. A clinical study evaluated the practicability of uproleselan treatment with mitoxantrone, etoposide, and cytarabine in R/R and ND AML [125]. Among the 54 patients with R/R AML receiving the recommended dose, the CR/incomplete CR (CRi) rate was 41%, and 52% of those with one prior induction regimen achieved a fair level of tolerability. Recently, Enjeti et al. revealed a signaling pathway involving epigenetic mechanisms underlying this phenomenon via the use of uproleselan and the hypomethylating agent 5-azacitidine in AML mouse models [150]. Along with uproleselan, the increase in AML adhesion to E-selectin stimulated by 5-azacitidine is reversed, and the survival of AML mice is improved.

However, some results are not ideal. In a phase Ib/II clinical trial of uproleselan with cladribine and LDAC in the treatment of secondary AML, the ORR was 11%, with 65% of patients presenting with grade 3 neutropenic fever [151]. As announced by GlycoMimetics in May 2024, in a phase III study of uproleselan combined with 3 chemotherapy drugs, namely, either MEC (mitoxantrone, etoposide and cytarabine) or FAI (fludarabine, cytarabine and idarubicin), in 388 patients with R/R AML, no statistically significant improvement in OS was achieved versus the administration of chemotherapy alone [152]. Genetic engineering research has shown that increased level of fucosylation on the surface of NK cells boosts their homing ability and increase immune cytotoxicity in the AML microenvironment [153]. This finding suggests a novel strategy for NK-based therapy while offering distinct insight into the potential impairment of the intensity of the immune response by E-selectin inhibition. To date, the role of E-selectin with its ligand in AML has not been fully elucidated.

### Non-phagocytic ICIs in AML

Despite their limited efficacy as single drugs, ICIs in combination therapies have shown versatile supplementary functions [154]. PD-1 blockade is considered to be a safe but inferior alternative for HSCT, achieving similar 3-year OS with higher mortality due to relapse than HSCT in non-favorable-risk AML patients in remission [155]. In combination with chemotherapy, PD-1 antibodies are effective in bridging HSCT in R/R AML patients with reduced incidence of GVHD [156]. Similarly, the anti-CTLA-4 mAb ipilimumab with decitabine is reported to be a practicable bridge to HSCT in MDS/AML patients [157]. As an IC expressed on multiple immune cells, LILRB1 blockade enhanced the NK cell-mediated cytotoxicity of multiple myeloma, leukemia, and lymphoma cells in vitro and in vivo [158]. T-cell immunoglobulin and mucin domain-3 (TIM-3) is a T cell IC that shows expression on AML blasts but not normal HSCs, making the blockade more effective in AML. Abdel-Rahman et al. constructed a pharmacophore-based screening technology and successfully identified TIM-3 small molecule inhibitors that suppressed the immune function of T cells in vitro [159]. While the research focus has been therapeutic approaches and indications targeting these ICs, durability should also be a matter of concern.

### Novel technologies for targeting AML blasts

**Fig. 3:**
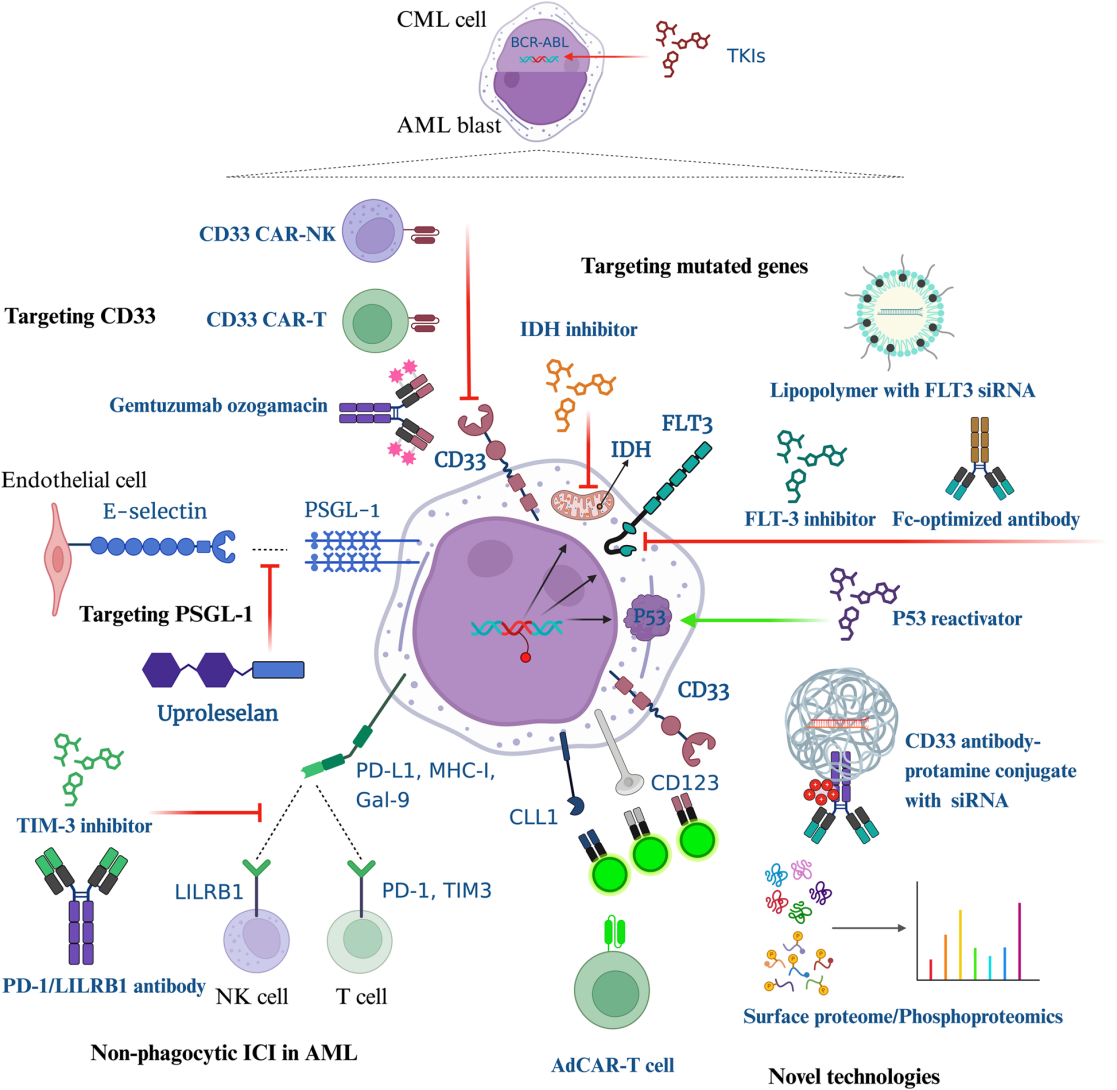
Targeting myeloid-derived blasts in myeloid malignancies. AML blasts present higher heterogeneity compared with CML cells, hence much more in need of diverse targeting therapies. Targeting BCR-ABL fusion gene for CML using TKIs is the major strategy. Strategies targeting AML blasts include targeting CD33, PSGL-1, mutated genes, and non-phagocytic ICI. Targeted mutated genes are FLT3, IDH and TP53 using inhibitor, antibody or reactivator etc. Non-phagocytic ICs currently known are TIM-3, PD-1 and LILRB1. Promising novel targeted strategies include AdCAR-T cell therapy, CD33 antibody-protamine conjugate with siRNA and surface proteome or phosphoproteomics

To identify feasible targets for myeloid blasts, technology empowers the field to a great extent. One promising approach is surface proteome and single-cell transcriptome analysis, which reveals antigens expressed by different subpopulations to construct blast marker expression profiles, guiding stratification in treatment and prognosis [160]. This study provides a comprehensive analysis of the AML surfaceome, identifying specific surface antigens preferentially expressed by primitive AML cells to enhance immunotherapeutic targeting. Borek et al. identified phosphoproteomic FLT3-mutated AML subtypes that can provide accurate prediction of sensitivity to midostaurin with chemotherapy, which contributes to the development of an FLT3-based stratification method [161]. Nixdorf et al. introduced adapter chimeric antigen receptor (Ad CAR) T cells in AML by offering adapter molecules that can bind with various surface antigens while linking to single CAR-T cell with the same epitope. This approach addresses T-cell exhaustion and antigen heterogeneity, with release intervals to avoid consistent exposure to antigens, which shows feasible target specificity and efficiency in AML [162]. Nanotechnology is another steadily developing approach where materials at nanometer scales are widely used for drug delivery and precise medicine. Zong et al. developed a multistage vector, enfolding parthenolide which can ablate LSCs, while embedded with E-selectin thioaptamer to selectively deliver the drug to endothelium in BM [163]. These nanoparticles successfully impaired BM protective niche and suppressed LSCs in vivo. Bäumer et al. introduced an electrostatic carrier containing DNA methyltransferase 3 alpha (DNMT3A) targeting small interfering RNAs (siRNAs), wrapped with anti-CD33 gemtuzumab attaching to cationic protamine. Selective transportation to blasts and oncogene-specific functionality of the conjugate were found to be effective in DNMT3A-mutated AML cells. The development of RNA interference (RNAi) and electrostatic binding enable the nanoparticle to be more precise and effective. As techniques emerge, novel approaches for pharmaceutical modification and delivery methods are expected to arise exponentially. The summary of strategies in myeloid malignancies targeting myeloid-derived blasts is drawn in Fig. 3.

## Conclusion and future perspectives

Among myeloid cells, macrophages seem to be of utmost importance in immunotherapy on the basis of the current research status [80]. Targeting polarization, phagocytosis and recruitment are major strategies for HMs, with ICIs at the forefront. For CD47, with several ongoing clinical trials, finding combination drugs with synergetic effects and eligible indications is key for the eradication of blasts while maintaining tolerance. Novel phagocytic ICs, such as CD24, along with other undiscovered molecules, should be studied for their potential therapeutic effects. Determinant genes or receptors that regulate macrophage polarization and recruitment are at an early stage of development and have relatively few clinical applications; however, they are still promising. CAR-Ms exhibit directed phagocytosis that depends on effective targets, which still has much to overcome, especially for HMs. Immunosuppressive MDSCs constitute a subgroup of emerging target cells for HMs, although the understanding of these cells is not comprehensive enough to identify effective clinical drugs. Targeting the formation, function and recruitment are developing approaches. Myeloid-derived blasts are the hardcore in HMs, and targeting mutated genes and altered surface biomarkers are primary therapeutic goals, whereas off-target toxicity and heterogeneity make target acquisition considerably intricate. The selection of suitable subjects for traditional targets and the identification of novel practical targets are needed. PSGL-1/E-selectin, which is involved in AML immune escape and NK cell homing, stresses the complexity of the immune environment of leukemogenesis. Technologies can guide progress throughout the process, from target identification to selection, from compound detection to modification, and from patient stratification to treatment management.

Although improvements have been made in the field, perspectives and some challenges still need to be addressed. (1) Few myeloid cell-based therapies are currently in the clinical field. How novel therapies can substitute or be an alternative for inadequate drugs and how to ease their way in standard treatment or find their position are yet to be determined. (2) For molecules such as CD47, finding a balance between therapeutic effects and AEs determines success in clinical trials, which still needs to be further tested. (3) Novel epitopes for recognition or function should be detected for their ability to be distinguished from normal blood cells. (4) Notably, macrophages are predominant in inducing CRS after CAR-T therapy in the treatment of various HMs [164]. Alleviation is focused on impairing macrophage activation without compromising CAR-T cell efficiency, suggesting that common AEs should be considered when they are applied together [165]. (5) The characterization of MDSCs and LSCs population remains incomplete, including how their cellular definitions relate to phenotypic expression, and the extent of their heterogeneity. A better understanding of these aspects is essential for selecting appropriate therapeutic targets. For common antigens expressed on MDSCs and blasts such as CD33 and CD123, it is necessary to investigate whether targeted therapy would be more effective [96, 97]. Researchers have not determined whether the origin of macrophages or the identification of MDSCs is relevant to their function, which might be crucial for achieving therapeutic goals [45]. (6) How emerging technology can be used to target myeloid cells for therapeutic approaches should be explored further. For instance, systemic multifunctional nanovaccines can induce strong antigen presenting ability of dendritic cells in vitro and long-lasting AML blasts eradication in vivo [166]. This tumor cell lysate-based platform is applicable to treat various HMs, which provides valid aspects for future investigation. (7) Inspite of the heterogeneity of AML blasts and the resilience of LSCs, the induction of certain phenotypical expression might provide effective and durable targeted effects. The surface proteomics have demonstrated a vast number of possible antigens, including CD38 [160]. However, CD38 shows unstable expression on AML cells and does not express on LSCs, causing targeted therapy to have minimum efficacy [167]. A single-chain CD38 T-cell engager managed to counter AML cells while stimulate T cells to release IFN-γ, which can induce CD38 expression on LSCs to be targeted as well, showing significant leukemic killing effects in vivo while sparing normal HSCs [168].

Myeloid lineage as a target has enormous prospects, whether it is dominative pathological blasts or a proportion of the grand immune environment. Just as the heterogeneity in cytogenetics and immunophenotype myeloid-derived blasts present, myeloid cells which act as the vital innate immune cells are diverse and versatile, with promising targets for initiating cytotoxicity and reversing immune evasion of malignant cells [8, 99]. New approaches to regulate cell function and deliver drugs to HMs are emerging, and with advancing techniques, it is expected that targeting myeloid cells for treating HMs has unlimited potential.

## Data Availability

No datasets were generated or analysed during the current study.

## Abbreviations

ADC: Antibody‒drug conjugate
ADCC: Antibody-dependent cell-mediated cytotoxicity
ADCP: Antibody-dependent cell-mediated phagocytosis
Ad CAR: Adapter chimeric antigen receptor
AEs: Adverse effects
AML: Acute myeloid leukemia
BM: Bone marrow
BsAb: Bispecific antibody
CAR-M: Chimeric antigen receptor-macrophage
CCL2: Chemokine ligand 2
CCR2: Chemokine receptor type 2
CDC: Complement-dependent cytotoxicity
CD47: Cluster of differentiation 47
cHL: Classical Hodgkin lymphoma
CLL1: C-type lectin-like molecule-1
CML: Chronic myeloid leukemia
CR: Complete response
CRS: Cytokine release syndrome
CSF-1: Colony stimulating factor 1
CTCL: Cutaneous T-cell lymphoma
CTLA-4: Cytotoxic T-lymphocyte antigen 4
DLBCL: Diffuse large B-cell lymphoma
DNMT3A: DNA methyltransferase 3 alpha
E-selectin: Endothelial adhesion molecule 1
FLT3: FMS-related tyrosine kinase 3
GM-CSF: Granulocyte macrophage-colony stimulating factor
GO: Gemtuzumab ozogamicin; GVHD:graft-versus-host disease
HER-2: Human epidermal growth factor receptor 2
HMs: Hematological malignancies
HR: High-risk
HRS: Hodgkin and Reed–Sternberg
HSCs: Hematopoietic stem cells
HSCT: Hematopoietic stem cell transplantation
ICs: Immune checkpoints
ICIs: Immune checkpoint inhibitors
IDH: Isocitrate dehydrogenase
IDO: Indoleamine 2,3-dioxygenase
IL-10R: Interleukin-10 receptor
ITD: Internal tandem duplication
iPSCs: Induced pluripotent stem cells
LAMs: Leukemia-associated macrophages
LILRB1: Leukocyte immunoglobulin-like receptor B1
LSCs: Leukemic stem cells
MARCO: Macrophage receptor with a collagenous structure
MCL: Mantle cell lymphoma
MDS: Myelodysplastic syndrome
MDSCs: Myeloid-derived suppressor cells
MerTK: MER proto-oncogene tyrosine kinase
MIF: Migration inhibitory factor
mAb: Monoclonal antibody
MM: Multiple myeloma
MMP8: Matrix metalloproteinase 8
MRD: Minimal residual disease
ND: Newly diagnosed
NHL: Non-Hodgkin lymphoma
NK: Natural killer
NKG2A: Natural killer cell receptor group 2 member a
OR: Overall response
ORR: Overall response rate
PB: Peripheral blood
PBMCs: Peripheral blood mononuclear cells
PD: Progressive disease
PD-1: Programmed cell death protein 1
PD-L1: Programmed cell death-ligand 1
PSGL-1: P-selectin glycoprotein ligand-1
PTCL: Peripheral T-cell lymphoma
RBCs: Red blood cells; RNAi:RNA interference
R/R: Relapsed/refractory
SD: Stable disease
Siglec-10: Sialic acid-binding immunoglobulin-like lectin-10
siRNAs: Small interfering RNAs
SIRPα: Signal regulatory protein α
S100A4: S100 calcium-binding protein A4
TAM: Tumor-associated macrophage
TEAEs: Treatment-emergent adverse effects
TIGIT: T-cell immunoreceptor with Ig and ITIM domains
TIM-3: T-cell immunoglobulin and mucin domain-3
TME: Tumor microenvironment
TNFα: Tumor necrosis factor α
TKIs: Tyrosine kinase inhibitors
TP53: Tumor protein 53
TREM2: Triggering receptor expressed on myeloid cells 2
